# Characterization of a PLLA-Collagen I Blend Nanofiber Scaffold with Respect to Growth and Osteogenic Differentiation of Human Mesenchymal Stem Cells

**DOI:** 10.1100/tsw.2009.13

**Published:** 2009-02-15

**Authors:** Markus D. Schofer, Ulrich Boudriot, Irini Leifeld, Romina I. Sütterlin, Markus Rudisile, Joachim H. Wendorff, Andreas Greiner, Jürgen R. J. Paletta, Susanne Fuchs-Winkelmann

**Affiliations:** ^1^ Department of Orthopedics, University of Marburg, D-35043 Marburg, Germany; ^2^ Department of Orthopedics, Sankt-Elisabeth-Hospital, Gütersloh, D-33332 Gütersloh, Germany; ^3^ Department of Chemistry, University of Marburg, D-35032 Marburg, Germany

**Keywords:** nanofibers, tissue engineering, human mesenchymal stem cells, PLLA, collagen I blend

## Abstract

The aim of this study was to enhance synthetic poly(L-lactic acid) (PLLA) nanofibers by blending with collagen I (COLI) in order to improve their ability to promote growth and osteogenic differentiation of stem cells *in vitro*. Fiber matrices composed of PLLA and COLI in different ratios were characterized with respect to their morphology, as well as their ability to promote growth of human mesenchymal stem cells (hMSC) over a period of 22 days. Furthermore, the course of differentiation was analyzed by gene expression of alkaline phosphatase (ALP), osteocalcin (OC), and COLI. The PLLA-COLI blend nanofibers presented themselves with a relatively smooth surface. They were more hydrophilic as compared to PLLA nanofibers alone and formed a gel-like structure with a stable nanofiber backbone when incubated in aqueous solutions. We examined nanofibers composed of different PLLA and COLI ratios. A composition of 4:1 ratio of PLLA:COLI showed the best results. When hMSC were cultured on the PLLA-COLI nanofiber blend, growth as well as osteoblast differentiation (determined as gene expression of ALP, OC, and COLI) was enhanced when compared to PLLA nanofibers alone. Therefore, the blending of PLLA with COLI might be a suitable tool to enhance PLLA nanofibers with respect to bone tissue engineering.

## INTRODUCTION

The reconstruction of large bony defects often requires the use of graft material. Modern tissue engineering offers an alternative for the treatment of critical bone defects. Artificial scaffolds based on synthetic biomaterials, such as metals, ceramics, polymers, and composites, have been developed for bone tissue engineering[1,2]. Among them, scaffolds based on nanofibers produced by electrospinning offer great advantages[3,4]. Besides the fact that these fibers mimic the extracellular matrix[5,6], they can be produced from a broad spectrum of biocompatible and biodegradable polymers[3,7]. With respect to bone tissue engineering, several groups have demonstrated that nanofibers or nanostructured materials are suitable in order to culture osteoblast-like cells or mesenchymal stem cells *in vitro*[8,9,10,11,12,13,14]. In the case of poly(L-lactic acid) (PLLA), we reported earlier that nanofibers made of this polymer did not interfere with growth and differentiation of human mesenchymal stem cells (hMSC), in principle[15]. Although these cells grew well on PLLA nanofibers, they showed an initial down-regulation of the expression of genes associated with osteoblast differentiation[16]. In order to overcome this, several strategies may be suitable. Substances that are capable of triggering osteogenesis must be included[17]. Besides growth factors like bone morphogenetic protein 2 (BMP-2)[18], the incorporation of type I collagen into the PLLA nanofibers might offer some advantages. It is known from other studies that the adhesion to collagen I (COLI) via the α2β1 integrin is sufficient to induce osteogenic differentiation of hMSC even in the absence of exogenous soluble stimuli[19,20]. Furthermore, we[16] and others[10] demonstrated that pure collagen nanofibers support the growth of hMSC without compromising their osteogenic differentiation capability. Nevertheless, the need for fixation of the collagen nanofibers and their lack of stability during cell culture may compromise their usage as a scaffold for bone tissue engineering. Therefore, this study was designed to combine the stability of PLLA nanofibers with the osteoinductive capability of COLI nanofibers by blending these polymers.

## MATERIAL AND METHODS

### Construction of Nanofibers and Characterization

The preparation of PLLA nanofibers by electrospinning has been reported in detail earlier[16,21]. In order to incorporate COLI into the nanofibers, PLLA and COLI were dissolved in hexafluoroisopropanol (HFIP) in the desired ratio, resulting in a 4.5% (w/v) polymer solution. The spinning process was performed at a flow rate of 0.2–0.3 µl/min, with an applied voltage of 10–18 kV and a distance of 15 cm. In order to establish an optimal PLLA-COLI scaffold, in some experiments we used different ratios of PLLA and COLI as indicated in the text. If the blend fibers were fixed, 4% glutaraldehyde was used over a period of 2 h. The residues were removed by extensive washing using phosphate buffer solution (PBS).

### Contact Angles

Static contact angles of water were measured using the sessile drop method with a G10 Drop Shape Analysis System (Krüss, Hamburg, Germany) and calculated using Data Physics SCA20 Contact Angle Analyzer Software.

### Scanning Electron Microscopy

For scanning electron microscopy (SEM), samples were sputter coated with platinum and examined in a SE microscope (SSM-7500F, SEOL Ltd., Tokyo, Japan) at an accelerating voltage of 2–5 kV in the SE mode.

## hMSC Isolation, Characterization, and Culture hMSC were obtained from consenting patients with the approval of the institutional review board. The indication for surgery was primary osteoarthritis of the hip with increasing pain, decreased range of motion, and signs of progressive osteoarthritis in radiographs. The patients had no evidence of other bone or autoimmune diseases. The routinely removed bone was obtained from the proximal femur while preparing the implant bed. hMSC were isolated and cultured according to the preparation of Pittenger et al.[22], with minor modifications as described by Brendel et al.[23].

Within the experiments, hMSC preparations negative for CD34 (CD, cluster of differentiation) and CD40, and positive for the stem cell markers CD90 and CD105, were used. In order to ensure the osteoinductive potential of the obtained cells, gene expression of alkaline phosphatase (ALP), in response to dexamethasone, was determined prior to the experiments.

For experiments, hMSC were seeded at a density of $3 \times 10^4$ cells/cm² on cover slips or cover slips coated with either PLLA or PLLA-COLI blend nanofibers in growth medium (Dulbecco's Modified Eagle Medium, DMEM), with low glucose and glutamine (PAA, Linz, Austria) supplemented with 10% fetal calf serum (FCS) from selected lots (Stem Cell Technologies, Vancouver, Canada) and 1% penicillin/streptomycin. In some experiments, osteogenic differentiation was induced according to Jaiswal et al. after an initial proliferation phase of 3 days[24]. The medium was generally replaced every 2$^{nd}$ day of culture.

## Gene Expression Analysis

RNA extraction, cDNA synthesis, and quantitative polymerase chain reaction (PCR) analysis were performed as described earlier[16,18,25]. Cycle temperatures and incubation times for human ALP, osteopontin (OP), collagen I (COLI), osteocalcin (OC), and 18s rRNA were previously described[25,26,27]. Purity of the single PCR products was verified by melting point analysis.

## Immunofluorescence Microscopy

Immunofluorescence analysis was carried out as described earlier[16,18]. Samples were fixed in acetone/methanol, washed, and exposed to blocking buffer at room temperature. Then cells were incubated with primary antibodies against COLI (Abcam, Ab6308, Cambridge, U.K.), OC (Acris, BP710, Hiddenhausen, Germany), and Ki-67 (Darko, Hamburg, Germany). Visualization was done, using a cy-2– or cy-3–conjugated secondary antibody (Dianova, Hamburg, Germany) at room temperature (1 h) and stained with 4',6-diamidino-2-phenylindole (DAPI). Fluorescence microscopy was performed using a Leica DM5000. Microphotographs of at least three different areas were made at a primary magnification of 20-fold high-power field (HPF). The intensity of fluorescence was determined using Quips analysis software. The total cell count of DAPI-stained nuclei was obtained. The proliferation index was calculated as a ratio of Ki-67 positive vs. total cells.

## Statistics

All values were expressed as mean ± standard error of at least three different patients and compared using students't-test or ANOVA (analysis of variance) with Bonferroni as a posthoc test. Values of $p < 0.05$ were considered to be significant. Significant values were marked with *, whereas $^{+}$ indicated a tendency within the patient series.

## RESULTS

### Characterization of PLLA-COLI Blend Nanofibers

SEM of electrospun PLLA-COLI blend nanofibers revealed a 3-D nonwoven network (Fig. 1) with a diameter of 445 ± 103 nm in the case of a blend ratio of 1:1, and 429 ± 64 nm for the blend consisting of four parts PLLA and one part COLI (4:1). The contact angle was 84 ± 13° (1:1 ratio) and 51 ± 8° (4:1 ratio). The PLLA-COLI blend nanofibers presented themselves with a relatively smooth surface devoid of pores. In aqueous solutions, the blend fibers formed a gel-like structure over time, with a stable nanofiber backbone for 22 days (Fig. 2). This backbone was not altered in the presence of cells and was independent of culture conditions. Furthermore, SEM studies showed evidence that cells grew well on the PLLA-COLI (1:1) blend (Fig. 1).

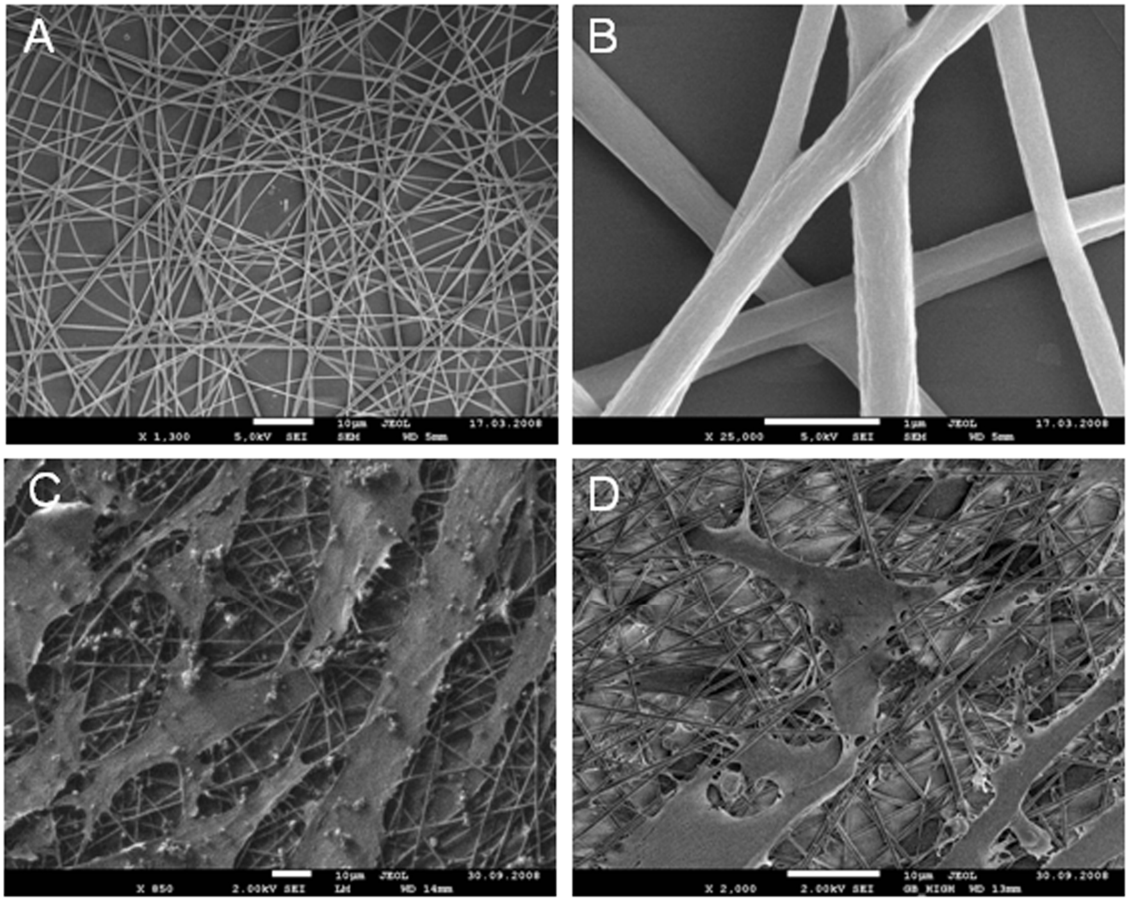

**FIGURE 1.** Characterization of PLLA-COLI blend. SEM analysis of a PLLA-COLI (1:1) blend (A,B). Same fibers after cell culture over a period of 22 days in growth medium (C) and differentiation medium (D).

### Finding the Optimal Blend Ratio

Based on findings that cells grew well on the PLLA-COLI (1:1) blend, different ratios of nanofiber blends were seeded with hMSC in order to find the optimal composition of PLLA-COLI blend fibers. After an incubation time of 22 days, cell densities and immunofluorescence staining of COLI and OC were analyzed.

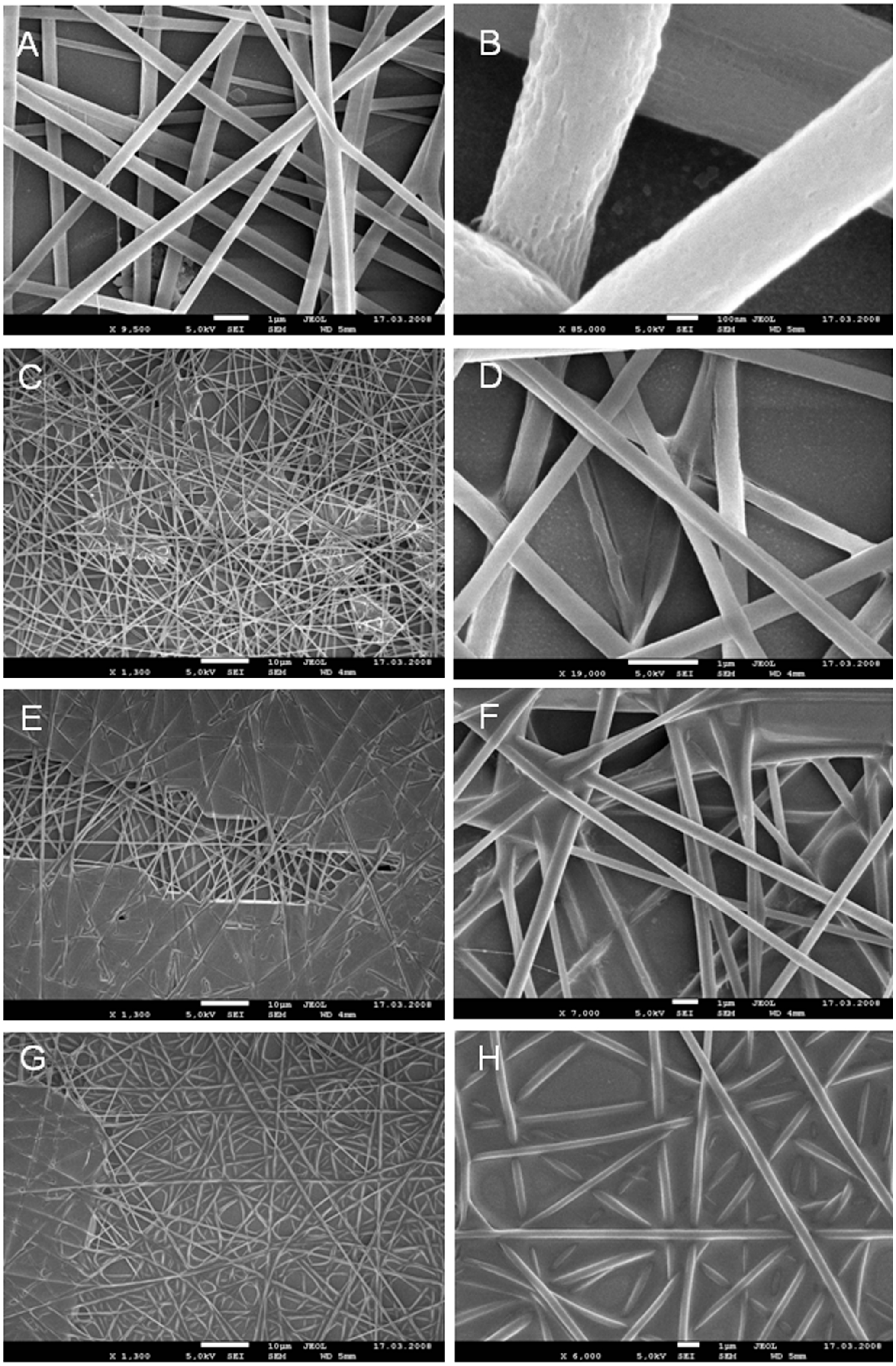

**FIGURE 2**. Long-term stability of PLLA-COLI blend nanofibers in aqueous solutions. SEM analysis of PLLA-COLI blends (1:1) after incubation in aqueous solvents over a period of 4 (A,B), 8 (C,D), 12 (E,F), and 16 (G,H) days. During the course of cultivation, a film formation can be observed, which results in a complete padding of the porous structure without compromising the PLLA fiber backbone.

Furthermore, in some experiments the blend nanofibers were fixed with glutaraldehyde in order to stabilize the fiber constructs (linked fibers). As shown in Fig. 3A, the highest cell densities were obtained when stem cells were cultured on unfixed blends. Cell densities depended directly on the blend ratio. Here, the composition of 4:1 PLLA:COLI seemed to be the most effective.

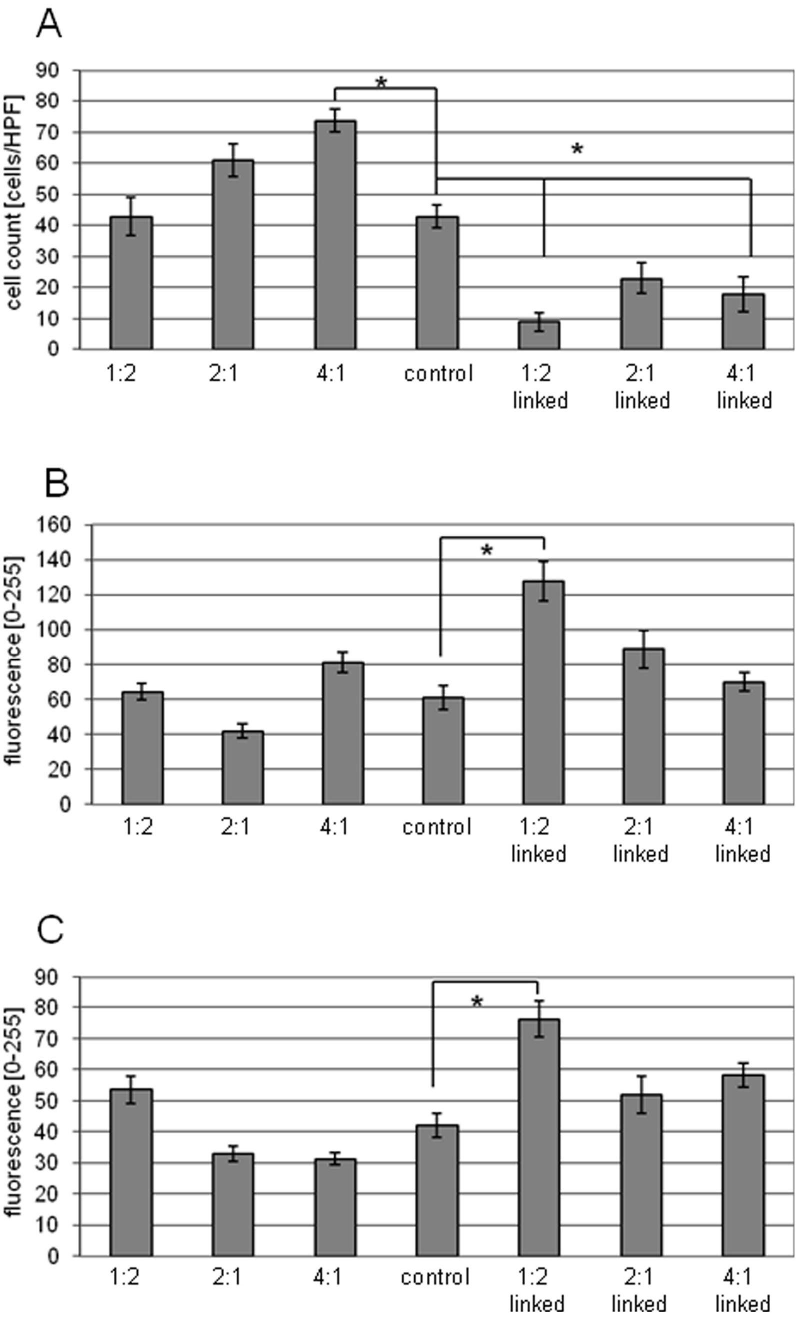

**FIGURE 3.** Influence of PLLA-COLI ratio on growth and differentiation of hMSC. Cell count (A), COLI deposition (B), and OC deposition (C) as determined by fluorescence analysis after 22 days of culture under growth conditions.

The fixation of the blend fibers generally resulted in a drastic decrease of cell densities compared to corresponding blends and controls. Nevertheless, the remaining cells stained stronger for collagen (Fig. 3B, Fig 4) and OC (Fig. 3C, Fig 4), especially when 1:2 PLLA:COLI blends were used.

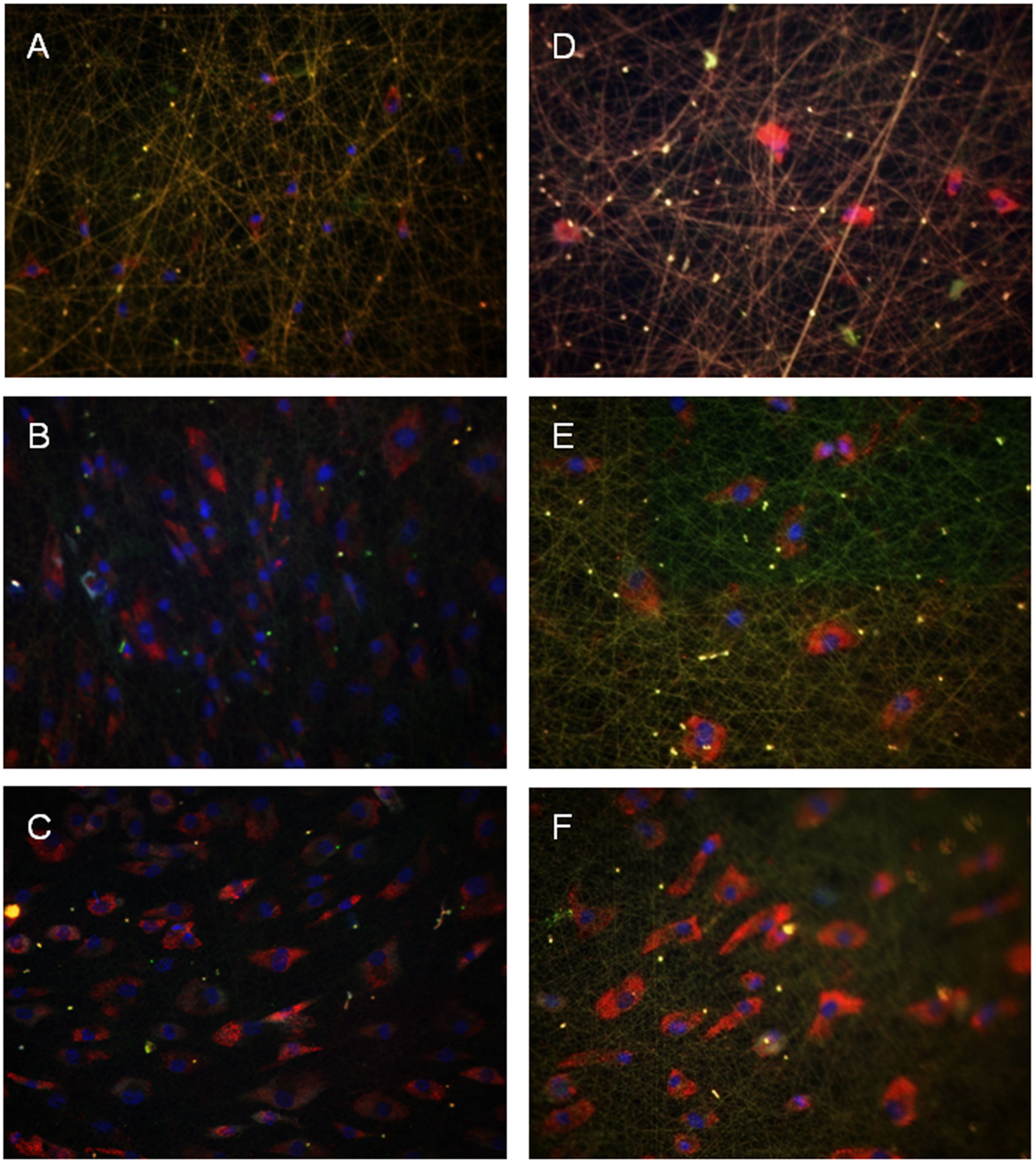

**FIGURE 4**. Influence of PLLA-COLI blending on growth and differentiation of hMSC. Immunofluorescence microphotographs of OC (green) and COLI (red) deposition of hMSC, cultured under growth conditions on PLLA-COLI blend nanofibers of different composition and fixation: PLLA:COLI 1:2 (A), PLLA:COLI 2:1 (B), PLLA:COLI 4:1 (C), PLLA:COLI 1:2 linked (D), PLLA:COLI 2:1 linked (E), PLLA:COLI 4:1 linked (F).

However, with respect to material properties and cell growth, the unfixed 4:1 PLLA:COLI blend seemed the most promising and therefore was used in further studies.

### Growth and Proliferation of hMSC Cultured on Nanofibers

In order to describe the biological effects, we analyzed the effect of PLLA-COLI blend nanofibers on the growth and proliferation of hMSC. These results were compared to the growth and proliferation of hMSC cultured on cover slips or PLLA nanofibers, respectively. Here, we used the unfixed 4:1 PLLA-COLI blend due to its ability to promote cell growth. As shown in Fig. 5A, there was no difference in the initial proliferation rate (day 4), determined by Ki67 staining, when cells were cultured under growth conditions. On the other hand, proliferation rates of stem cells on PLLA-COLI blend nanofibers were significantly elevated under osteoinductive conditions compared to growth on pure PLLA nanofiber and cover slips alone (Fig. 5A). Nevertheless, it seemed that the initial proliferation rate did not play an important role during growth of hMSC on PLLA-COLI blend nanofibers due to the fact that we detected higher cell densities on this fiber type during the course of cultivation (Fig. 5B,C).

### Influence of PLLA-COLI Blend Nanofibers on the Differentiation and Matrix Formation of hMSC

To examine whether blending of PLLA nanofibers with collagen is a suitable tool to enhance the osteoinductive properties of PLLA nanofibers, and to analyze the impact on hMSC differentiation towards osteoblasts, we compared the expression levels of ALP, COLI, and OC of cells grown on PLLA with cells grown on PLLA-COLI blend nanofibers (Fig. 6). Regardless of whether the cells were cultured under growth or osteoinductive conditions, the PLLA-COLI blend nanofibers showed an increase in osteoblast marker gene expression compared to the PLLA fibers (Fig. 6). This effect was especially evident in the early cultivation.

## DISCUSSION

PLLA is a biocompatible, biodegradable, and Food and Drug Administration–approved polymer; commonly used as pins, screws, or membranes; in bone reconstructive surgery[28,29,30,31]. As reported earlier, PLLA can easily be electrospun to a 3-D nonwoven network[32,33]. These constructs are appropriate as a matrix for osteoblast growth as well as for the osteogenic differentiation of hMSC in principle[15,16,21,34], although differentiation is diminished during the initial phase of cultivation[16]. In order to enhance the osteoinductive properties of PLLA nanofibers, the blending of the PLLA with COLI seems to be a suitable tool due to the osteoinductive properties of collagen nanofibers[10,16].

Indeed, the blending of PLLA nanofibers with COLI resulted in a decrease in hydrophobicity compared to PLLA nanofibers alone[16]. Moreover, the detected water contact angles of the blend nanofibers were similar to those of collagen nanofibers independent of the composite of the blend[16]. Similar findings were made concerning the fiber diameter, which was approximately half of the diameter obtained from PLLA nanofibers electrospun from dichloromethane[32] and comparable to the fiber diameter of COLI nanofibers[16]. Regarding these parameters, the PLLA-COLI blend fibers possessed similar properties to collagen nanofibers. Furthermore, they showed obvious similarities to the PLLA-gelantine blends studied by Hae-Won et al.[35]. Although the blend formed a gel-like structure, blend fibers had a stable backbone even in the presence of cells. Therefore, the blend possessed a higher stability compared to thermal cross-linked COLI nanofibers alone[16].

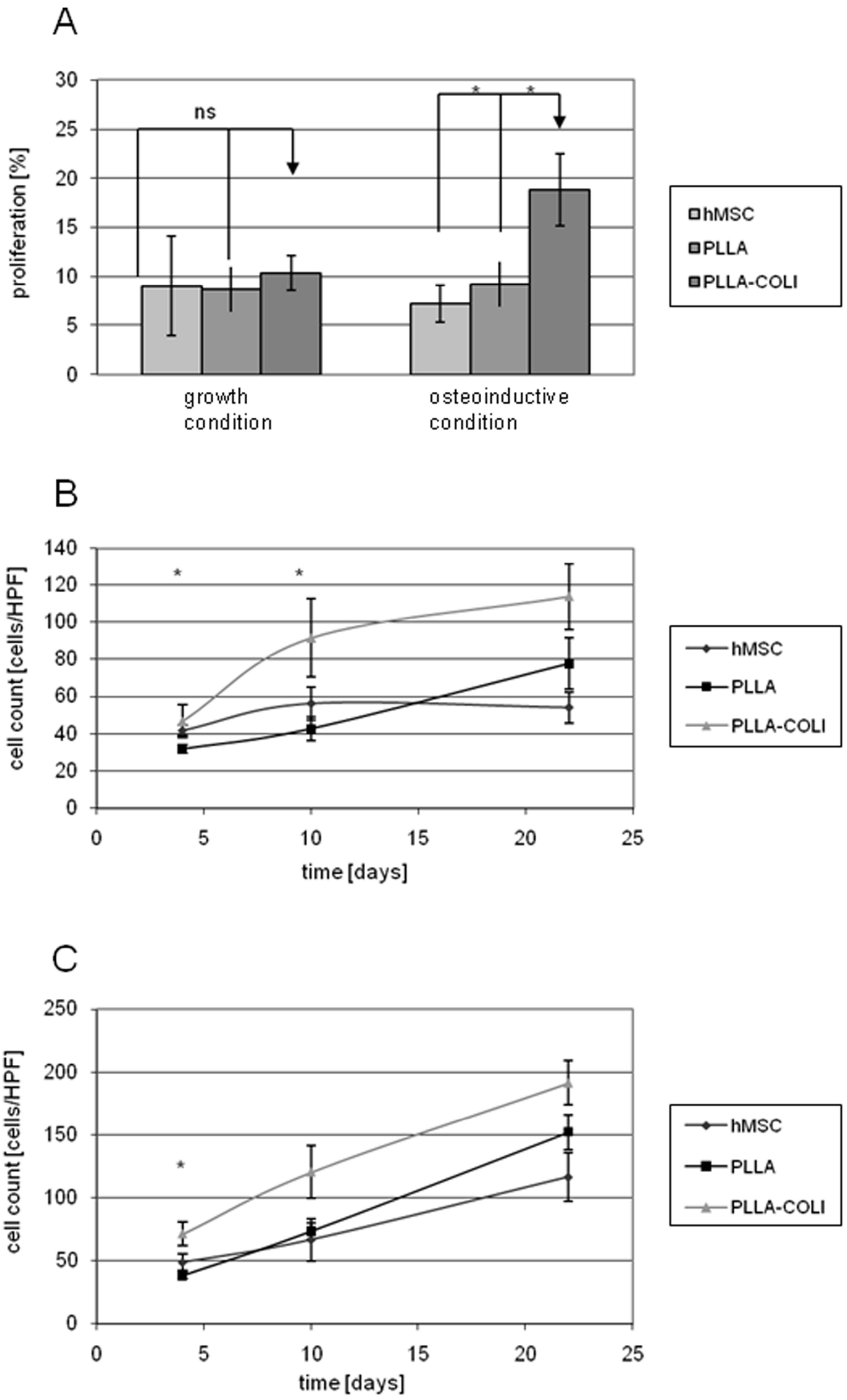

**FIGURE 5**. Influence of PLLA-COLI (4:1) blend on growth and proliferation. Proliferation of hMSC cultured under both growth and osteoinductive conditions at day 4 (A). Time course of cell densities cultured under growth (B) as well as osteoinductive conditions (C). (hMSC – control group hMSC cultured on cover slips.)

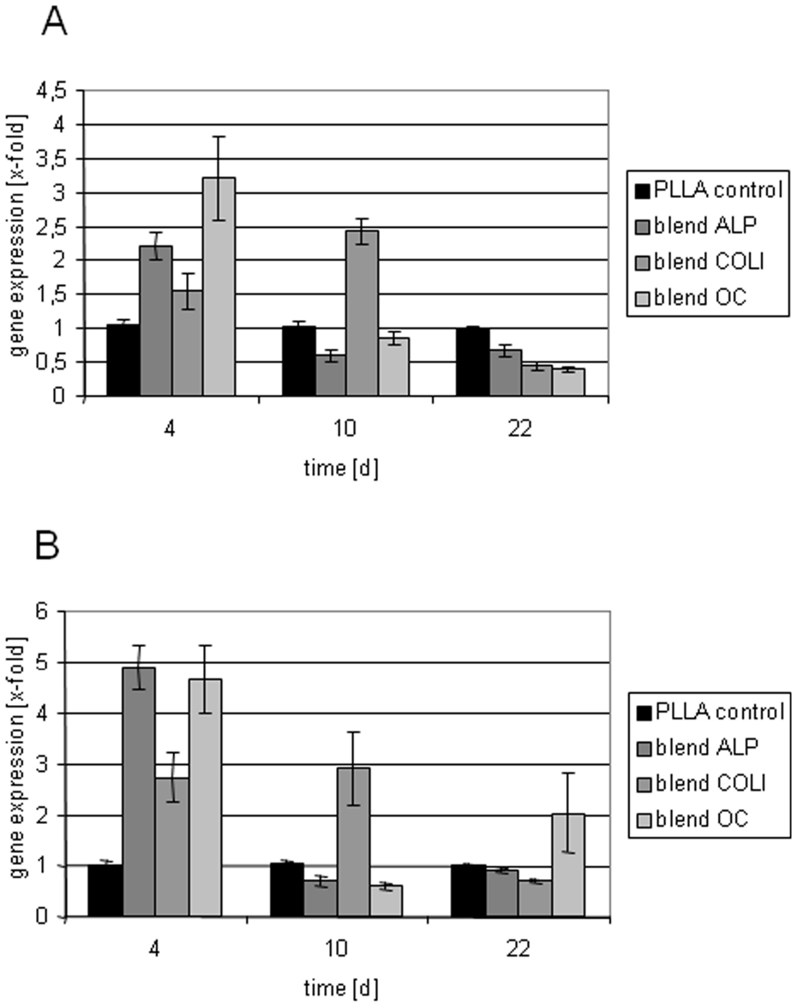

**FIGURE 6.** Influence of PLLA-COLI (4:1) blend on gene expression. Time course of gene expression of hMSC cultured under growth (A) and osteoinductive conditions (B), on PLLA-COLI blend nanofibers (blend) compared to PLLA nanofibers alone (PLLA control).

Focusing on the biocompatibility of blend nanofibers with different PLLA:COLI ratios, we found higher cell densities when hMSC were cultured on blend fibers compared to PLLA nanofibers or cells cultured on cover slips. The observed effect depended on the composition of the blend. With respect to growth, a composition of 4:1 ratio of PLLA:COLI showed the best results. Growth of stem cells was more similar to growth obtained on collagen fibers[10,16] and comparable to growth obtained on PLLA-gelantine blends[35]. Therefore, the inhibitory effect of PLLA polymer[36,37] and PLLA nanofibers[38] on cell densities was equalized. However, the increase in cell densities cannot be explained exclusively by an enhanced proliferation rate. The observed discrepancy might be explained by the fact that blend nanofibers provide an optimal 3-D surface for cell attachment. The collagen component might provide a cell adhesive surface, including RGD-integrin interactions. As a consequence, higher cell densities could be observed compared to PLLA nanofibers or glass surfaces.

When hMSC were cultured on blend nanofibers, we found an increase in the expression of genes associated with osteoblast lineage compared to pure PLLA nanofibers. Admittedly, this effect was confined to the early phase of culture. Although we have no data comparing the gene expression of hMSC cultured on the PLLA-COLI blend with that of glass surfaces, it is likely that blending with COLI prevents the inhibitory effect of the PLLA in the early phase of cultivation, at least to some extent[16].

Furthermore, the blend fibers were sufficient to induce osteogenic differentiation of hMSC even in the absence of exogenous soluble stimuli. This might be due to the adhesion to COLI[19,20].

However, the differentiation behavior of hMSC is different from that obtained on thermal cross-linked nanofibers, where the osteogenic potential of the collagen was most obvious in the late stage of cultivation[16]. The reason for this is not clear, but it might be associated with the fixation and composition of the PLLA-COLI fibers. Although we did not analyze the gene expression of hMSC on glutaraldehyde-fixed blend fibers in detail (due to the extreme inhibitory effect observed on cell densities) the fluorescence analysis of the surviving cells pointed out that these cells deposited more OC as well as COLI within the extracellular matrix. In this case, the stabilization of the PLLA-COLI blend nanofibers may play an important role for their use in tissue engineering.

This is an *in vitro* study. How far these results can be assigned to *in vivo* conditions is currently being investigated in an animal experiment.

## ACKNOWLEDGMENTS

This work was supported by the Deutsche Forschungsgemeinschaft (German Research Foundation, Grant No. BO 3065/1-1).
